# Creating space for Indigenous healing practices in patient care plans

**DOI:** 10.36834/cmej.70095

**Published:** 2020-08-06

**Authors:** Ishan Poudel, Binay Chaudhary

**Affiliations:** 1 California Institute of Behavioral Neurosciences and Psychology, California, USA

We were delighted to read the article “Creating space for indigenous healing practices in patient care plans.”^1^

This article has thoughtfully advocated for the use of Indigenous healing practices in different parts and forms including as an alternative medicine in certain sections.

The authors have clearly advanced the idea of helpful integration of indigenous healing practices into the practice of modern medicine.

They have expressed the importance and feasibility of integrating indigenous healing practices with due consideration of the perception of physicians and identifying barriers to support this aspect of patient-centered care for Indigenous patients. We commend the effort of the authors, and believe this model of study can be replicated in larger geographical sections to gather information not only about the indigenous healing practices but also about other systems of medical practices (such as Naturopathy and Aboriginal traditional healing in parts of Australia).

On behalf of like-minded clinicians, we would like to thank the authors for broadening the horizon of medical practice and opening up new aspects to enhance and explore medical science.
